# Meat in the Diet: Differentiating the Benefits and Risks of Different Types of Meat

**DOI:** 10.3390/foods12122363

**Published:** 2023-06-14

**Authors:** Carlotta Giromini, D. Ian Givens

**Affiliations:** 1 Department of Veterinary Medicine and Animal Science, University of Milan, 20133 Milano, Italy; 2 CRC, Innovation for Well-Being and Environment (I-WE), Università degli Studi di Milano, 20122 Milano, Italy; 3 Institute for Food Nutrition and Health, University of Reading, Reading RG6 6EU, UK; d.i.givens@reading.ac.uk

The present Special Issue features three broad areas related to meat: meat and human health, the effects of animals’ diets on the nutritional characteristics of meat, and consumers’ attitudes about buying and consuming cell-based meat. The first two areas are related, whereas the third raises important consumer concerns about new, alternative technologies for meat production.

Although meat has featured in the human diet for centuries and has been an important source of high-quality protein and several other nutrients, concern is increasing that meat, particularly certain types of meat, may have detrimental impacts on health, especially when intake is high over a lifespan. Two papers in this Special Issue focus on the association between meat consumption and health. Geiker et al. [1] initially focused on the advice from the World Health Organization (WHO) and other bodies to restrict the intake of saturated fat, of which ruminant meat is a relatively rich source. They highlight that recent evidence from meta-analyses of both prospective studies and randomized controlled trials (RCTs) do not support the traditional view that saturated fats are linked to increased cardiovascular diseases (CVDs) or diabetes. Thus, meat consumption should not be restricted because of saturated fat, although some studies suggest that saturated fat from meat is associated with a higher risk of CVDs than that from dairy foods.

Since around the year 2000, the regular updates on the associations between food and cancer risk produced by the World Cancer Research Fund and others have consistently indicated a higher risk of colorectal cancer associated with processed meat compared with red meat. Geiker et al. [1] discussed the subject of the definition of processed meat. They suggested that the definition of processed meat is inconsistent with variation between studies and internationally; in some studies, the definition of red meat includes processed meat. Moreover, they highlight that in many private households and catering facilities, frying and grilling are typical processing methods, which may contribute to carcinogenic compounds although this would be associated with a lower risk than from industrially processed meat. A key conclusion is the need to standardize the definition of red, processed, and unprocessed meat products and for suitably powered RCTs with biomarkers to identify the type of meat consumed.

In a second review on meat and health [2], the authors examined the balance of the health benefits and risks of meat and processed meat consumption during key life processes and the association between meat consumption and a range of noncommunicable diseases. The review highlights that between 1961 and 2018, world meat production increased from some 60 to 300 Mt, with most of the increase in pork and poultry meat and in Asia. This indicates that meat products now constitute the major source of proteins in many developed countries. The review discusses the role of red meat as a source of heme iron which has higher bioavailability than the nonheme iron typically found in plant-based foods but also exists in animal-based foods. The WHO reports that about 30% of the world’s population suffers from anemia, much of which is due to iron deficiency. Some countries have a particularly high prevalence of anemia, such as India, where 60% and 40% of children and young women are anemic, respectively. Meat consumption in India has been traditionally low, but red meat as an iron source should be specified for children and young women. The practicalities of this would be complex. The evidence of very sub-optimal iron intake by U.K. female adolescents is also discussed, which highlights the substantial reduction in red meat consumption by this group over recent years.

The focus of the review was understanding the relative impact of meat type on the association with CVDs, type 2 diabetes, certain cancers, and dementia. In relation to CVD risk, the evidence from meta-analyses shows an increased risk associated with processed meat, which is generally higher than that for unprocessed red meat. White meat has a neutral association, although the amount of data is less than for red and processed meat. The finding for white meat is important as it is consumed in larger amounts than other meat, although more studies are needed. The prospective study findings on the association between meat consumption and type 2 diabetes indicate a higher risk with processed meat than with red meat; although, as for CVD, the findings are mixed. For example, a recent umbrella study of prospective cohort studies found no association of red meat with risk of type 2 diabetes, although a substantially increased risk was found for processed red meat (+44%) and processed meat in general (+37%).

The risk of colorectal cancer from processed meat consumption is approximately double that for red meat according to the World Cancer Research Fund/American Institute for Cancer Research (WCRF/AICR) ongoing review of the evidence. A recent meta-analysis of 148 studies broadly agrees with the WCRF/AICR findings, although some studies were likely used in both meta-analyses. This study also indicated a positive association of red meat and processed meat with breast cancer risk, a topic that needs more investigation. The review [2] in this Special Issue also reports on a recent cohort study of 490,000 subjects in the U.K. Biobank and highlighted that each increment of 25 g of processed meat per day was associated with 44% and 52% increased risks of dementia and Alzheimer’s disease, respectively. Unprocessed red meat and white meat showed no significant association. This is the only U.K. cohort study concerning meat consumption and dementia; however, given the increase in dementia prevalence, this study may be quite important, although more studies are needed.

Both reviews [1,2] concerning meat and health highlight the issues concerning the definition of processed meat and the limited high-quality data in some important areas. Given the potentially serious outcomes linked to CVDs, diabetes, cancer, and dementia the need is urgent for the uncertainties to be resolved along with improved evidence on the mechanisms involved.

Three papers in this Special Issue focus on the impact of the animal’s diet on the nutritional characteristics of the meat. In their review, Davis et al. [3] started by reminding the reader that ruminant animal beef production has been the subject of considerable debate and criticism owing to being an important source of methane, which is a potent greenhouse gas. They also point out that in the U.K. at least, reduction in red meat consumption is the most likely food product to be considered in relation to health and, by implication, more action is required to improve the nutritional quality of beef. In this review, the authors examined the nutritional benefits (authors’ words) of fatty acids in beef from organic and grass-fed beef animals compared with those of animals in intensive and conventional systems. Broadly, and as previously shown, increasing the amount of fresh forage in the animals’ diet increases the proportion of polyunsaturated fatty acids in the meat, predominantly due to an increase in α-linolenic acid (18:3, n-3), although beef from animals fed grass only exhibited a small but significant increase in the long chain n-3 fatty acids EPA, DPA, and DHA. Using three different dietary patterns, the authors found for all three patterns that the dietary intake of α-linolenic acid and EPA + DHA was lowest when beef was from an intensive system, notably higher when from an organic system, and highest when from a grass-fed system. However, the estimated fatty acid intakes were much more influenced by the human dietary pattern (i.e., the amount of beef consumed) than the feeding system used to produce the beef.

Overall, the grass-fed beef can influence the total diet n-3 fatty acid intake, but its impact is weak unless coupled with a diet that does not considerably restrict beef consumption.

Haug et al. [4] report the results of a human RCT that compared the consumption of 300 g/day, for six days, of beef produced from bulls that had been fed a diet supplemented with additional vitamins D, E, and K; n-3 fatty acids; and an organic yeast form of selenium with the consumption of beef from animals that did not receive the supplement. Full details of the animal work were provided in an earlier paper [5]. The aim was to assess if consuming the enriched meat by young women would lead to meaningful increased intake of nutrients that are often consumed in suboptimal amounts. This aim was also connected with the fact that the nutrients in meat such as selenium and some vitamins are more bioavailable than when supplied by plants. The enriched beef was slightly higher in selenium (12.6 µg/100 g) than the regular beef (10.0 µg/100 g), with small increases in vitamins D3 and E, although vitamin K (form MK4) levels were substantially higher. Although no significant increase was found in the in calculated selenium intake with the consumption of enriched beef, the serum selenium concentration increased from the subjects’ normal values if they were less than 85 µg/L.

Overall, the impact of the consumption of the beef from the animals receiving the nutrient supplement appeared to be small, possibly except for benefits for subjects with a low selenium and vitamin D status. The efficiency of the transfer of nutrients from the animals’ diet into meat is likely low, and the use of direct human dietary supplements would be more efficient and less costly.

Ribeiro et al. [6] reviewed the effect of seaweeds and their derived compounds in the diets of pigs and poultry on their meat quality and nutritional value. The review evidences the benefits of using seaweed in terms of meat oxidation reduction and shelf-life improvement. For pork meat in particular, seaweed may indirectly influence lipid peroxidation via the modulation of the gut microbiome in pigs. In the case of poultry, despite potentially contributing to reducing ammonia emissions, the responses of different poultry and algae species are factors to consider when using seaweed as feed supplements, given the high heterogenicity and chemical composition of seaweeds. The authors stated that the number of publications on this topic is limited compared with that on other alternative feed ingredients. The widespread use of this rich source of biomass is currently limited by the high cost and by the presence of antinutritional factors. The negative digestive implications of feeding seaweeds should be considered in future research to maximize their beneficial effects.

A manuscript in this Special Issue focuses on a subject of considerable scientific and social media interest: the introduction of cell-based meat into the market [7]. In particular, the authors present a survey of Brazilian consumers’ attitudes toward cultured meat. Of 4471 participants, 46.6% were keen to consume cultured meat in the future, considering it a promising and acceptable technology. Most respondents were keen to pay much less for cultured meat than for conventional meat, and only 4.8% were willing to pay more. The survey reports factors such as age, job, monthly income, and sex as impacting consumers’ vision. Even though opinion surveys should be interpreted with caution, say the authors, with the large number of participants, the study provides a valuable overview of consumer’s feelings. The results might be important for stakeholders and meat companies aiming to enter the cultured meat market in Brazil and may influence their development. Finally, the survey can be a model to be reproduced in the U.S. and European countries to assess consumer perception, which will be important for U.S. and EU companies keen to enter the cultured meat market.

Finally, the future of meat production and consumption will be driven not only by the meat type (e.g., animal species, rearing conditions, and meat processing) but also by the nutritional and functional characteristics associated with these products and by the novel technologies that may enable alternative and sustainable production. These alternative technologies may play a role in modulating the production of processed meat products (e.g., with improved nutritional characteristics), having an important impact on human health.

Overall, the papers cover the area of meat consumption and human health, the effects of animals’ diets on the nutritional and quality characteristics of meat, and consumers’ attitudes toward buying and consuming cell-based meat. These topics represent important areas for future research, and the data reported in this Special Issue can guide the activities of meat companies, farmers, and meat retailers.

Finally, we would like to thank the authors for their contributions in the present Special Issue. These provide an excellent vision of the various approaches needed for an understanding of the nutrition and health characteristics of meat and the factors that influence people’s decisions about its consumption.
